# Well-being through the lens of the internet

**DOI:** 10.1371/journal.pone.0209562

**Published:** 2019-01-11

**Authors:** Yann Algan, Fabrice Murtin, Elizabeth Beasley, Kazuhito Higa, Claudia Senik

**Affiliations:** 1 Sciences-Po, Paris, France; 2 OECD Statistics and Data Directorate, Paris, France; 3 CEPREMAP, Paris, France; 4 Hitotsubashi University, Tokyo, Japan; 5 Paris School of Economics, Paris, France; University of Warwick, UNITED KINGDOM

## Abstract

We build models to estimate well-being in the United States based on changes in the volume of internet searches for different words, obtained from the Google Trends website. The estimated well-being series are weighted combinations of word groups that are endogenously identified to fit the weekly subjective well-being measures collected by Gallup Analytics for the United States or the biannual measures for the 50 states. Our approach combines theoretical underpinnings and statistical analysis, and the model we construct successfully estimates the out-of-sample evolution of most subjective well-being measures at a one-year horizon. Our analysis suggests that internet search data can be a complement to traditional survey data to measure and analyze the well-being of a population at high frequency and local geographic levels. We highlight some factors that are important for well-being, as we find that internet searches associated with *job search*, *civic participation*, and *healthy habits* consistently predict well-being across several models, datasets and use cases during the period studied.

## Introduction

There is increasing demand to use measures of well-being in order to move beyond the classical income-based approach to measuring human development and progress [1]. GDP does not measure non-market social interactions, such as friendship, family, happiness, moral values or the sense of purpose in life. Subjective, self-reported, measures of well-being attempt to capture these dimensions through answers to questions such as: “All things considered, how satisfied are you with your life as a whole those days?” Economists are exploring the use of subjective well-being variables as a direct measure of utility [2]. Political leaders have embraced this move by calling for representative surveys of well-being, for example the EU-wide Survey on Living Conditions, which in 2013 included a module on well-being, and the resources devoted to measuring well-being at the Office for National Statistics in the United Kingdom. However, subjective well-being measures still present a number of challenges and concerns both in measurement and interpretation [3–6]. More recently developed measurement approaches, such as the Day Reconstruction Method [7,8], Experience Sampling Model [9] and Time Use Surveys [10] have helped to improve interpretation and understanding of subjective well-being.

This paper examines whether changes in internet search volumes over time can be used to model changes in subjective well-being over time, and to estimate well-being at frequencies and granularities difficult to obtain using survey data. We also examine whether these models can (within limits) give us insight into the factors underlying well-being by identifying the types of searches that are most highly and consistently related to well-being over the time period 2008–2013.

This paper builds on the recent literature that uses big data in social science research—while the capacity to collect and analyze massive amounts of data has transformed the fields of physics and biology, such progress has been slower in social sciences [11]. This gap began to be remedied with the early contribution of Ettredge et al. [12], which used internet search data to forecast the unemployment rate in the US. The same idea was explored by Askitas and Zimmermann [13], D’Amuri and Marcucci [14] and Suhoy [15], while Baker and Fradkin [16] use a measure of job search based on Google search data to study the effects of unemployment insurance and job finding. Choi and Varian [17,18] have explained how to use search engine data for forecasting macroeconomic indicators of unemployment, automobile demand, and vacation destinations, while several papers have analyzed consumer sentiment [19–21]. Regarding subjective well-being, Stephens-Davidowitz and Varian [22] used Google data to study trends of depression and anxiety. Stephens-Davidowitz [23] used Google Trends data to examine the role of racism in the 2008 and 2012 presidential elections. Closer to us, Schwartz et. al. [24] developed techniques that predict life satisfaction of Facebook users based on natural language. Finally, MacKerron and Mourato [25] use geo-spatial data to study subjective well-being in different physical environments.

Large datasets collected from sources such as Google, Twitter and Facebook appeal to social scientists because they allow researchers to observe people’s behavior directly rather than relying on what people say their behavior is. These data are also timely, generally available at a local level (as long as internet penetration and use is sufficient to obtain statistical representativeness), and available at low cost relative to surveys. Despite their attractive qualities, however, these data present a number of challenges, and this paper proposes methodological solutions to some of the issues in the Google Trends data. More generally, however, the volume of internet searches to be treated is potentially enormous, and it is a challenge to disentangle signal from noise while avoiding cherry picking. The Google Flu Trends is a well-known case where internet searches were matched with “small” data (as we do in this paper) and the model initially performed well but lost predictive power over time [11]. A likely cause was changes in search activity and the interface of Google Search itself (for example, auto-suggestion). Since this difficulty is structural to the Google Trends data, the accuracy of the estimates derived from Google Trends using our framework will depend on periodic updating and revision (this is also important to allow the model to incorporate social changes that could necessitate a re-weighting of the components).

Following the recommendations of Monroe et al. [26], we also combine theory and data analysis, drawing from the literature on subjective well-being, instead of applying algorithms to large datasets agnostically. Our methodology allows us to construct a model that has four important qualities: it is grounded in theory and the existing literature on well-being, it is testable and has strong out of sample performance, it is simple and transparent, and it is adaptable and can potentially be used to estimate well-being on a continuous and recurrent basis to examine the impact of shocks on well-being.

We find that searches related to job search, civic engagement, and healthy habits are the most consistently important predictors of well-being across different samples. We provide two examples of the way fluctuations in estimated well-being can be used to better understand responses to events: the decline in well-being in red states following the election of Obama and the change in well-being during the months after a mass layoff announcement.

## Results

We construct models that estimate well-being measures in the United States using a very large amount of search engine data covering the years 2008–2013. Rather than using search volumes for hundreds of words, data are condensed into several “composite category” variables that can be interpreted as different life dimensions (such as family life or financial stress). Using these composites we built two models that fit the Gallup survey trends in subjective well-being at the aggregate US level (forming the ‘US-level model’) as well as at the state level (yielding the ‘state-level model’). Both models display high out of sample correlation. We run a simple variance decomposition to quantify the contributions of each dimension to the estimated well-being. We also compare selected predictors across models, and find that, in particular, searches related to *Job Search*, *Civic Engagement*, and *Healthy Habits* have high predictive power for well-being across models and samples.

### Modeling subjective well-being in the United States

Table 1 (Life Evaluation and Positive Affects) and Table 2 (Negative Affects) present the results from the US-level model for each subjective well-being variable using either monthly or (more parsimoniously) quarterly time dummies at the national level and for the most part the models are quite similar (the dependent and explanatory variables have been standardized to allow for a comparison of the magnitude of the coefficients). The selected variables are, in general, not very sensitive to the selection procedure, and the coefficients are generally consistent for positive and negative affects. Categories of words that are consistently associated with higher well-being at the aggregate US level (excluding the Learn and Respect subjective well-being variables, for which our model does not perform well, as discussed below) are *Job Market*, *Civic Engagement*, *Healthy Habits*, *Summer Leisure*, and *Education and Ideals*. Categories that are consistently associated with lower well-being are *Job Search*, *Financial Security*, *Health Conditions*, and *Family Stress*.

**Table 1:** Regression of categories on subjective well-being data–life evaluation and positive affects (national level).

	Life evaluation today		Life evaluation in 5 years		Happiness		Laugh		Learn		Respect	
	(1)	(2)	(3)	(4)	(5)	(6)	(7)	(8)	(9)	(10)	(11)	(12)
**Material conditions**												
Job Search	-0.809***	-0.728***		-0.209**	-0.210***	-0.224***	-0.319***	-0.328***	-0.444***	-0.466***	-0.369***	-0.270***
	(0.047)	(0.077)		(0.086)	(0.077)	(0.068)	(0.066)	(0.060)	(0.065)	(0.061)	(0.056)	(0.087)
Job Market	0.608***	0.664***							-0.222***	-0.297***	1.218***	1.406***
	(0.130)	(0.131)							(0.081)	(0.101)	(0.070)	(0.148)
Financial Security		-0.390***	-0.954***	-1.176***	-0.437***	-0.494***	-0.366***	-0.385***	-0.357***	-0.415***		-0.495***
		(0.118)	(0.118)	(0.122)	(0.114)	(0.096)	(0.087)	(0.104)	(0.077)	(0.088)		(0.133)
**Social**												
Family Life					0.225***	0.359***	0.208***	0.259***	0.425***	0.403***		
					(0.085)	(0.083)	(0.057)	(0.063)	(0.052)	(0.057)		
Family Stress	-0.408***	-0.336***	0.463***	0.627***					-0.135**			
	(0.050)	(0.094)	(0.137)	(0.133)					(0.067)			
Civic Engagement	0.259**	0.557***					-0.341***	-0.346***				
	(0.105)	(0.124)					(0.104)	(0.131)				
Personal Security	0.199***	0.218***	-0.411***	-0.325***			-0.266***	-0.297***				
	(0.029)	(0.049)	(0.060)	(0.064)			(0.059)	(0.051)				
**Health and Wellness**												
Healthy Habits	0.207***	0.190**	0.782***	0.618***	0.579***	0.479***	0.618***	0.566***	0.243***	0.267***		
	(0.063)	(0.079)	(0.089)	(0.083)	(0.069)	(0.066)	(0.076)	(0.072)	(0.061)	(0.066)		
Summer Leisure									0.226**		-0.969***	-0.487**
									(0.089)		(0.071)	(0.239)
Health Conditions			-0.508***				0.594***	0.330**	0.443***	0.478***		
			(0.163)				(0.146)	(0.132)	(0.083)	(0.113)		
Education and Ideals	-0.257***	-0.306**	0.272**		-0.387***		-0.538***				-1.003***	-0.666***
	(0.043)	(0.147)	(0.117)		(0.079)		(0.095)				(0.085)	(0.199)
Time dummies	Quarterly	Monthly	Quarterly	Monthly	Quarterly	Monthly	Quarterly	Monthly	Quarterly	Monthly	Quarterly	Monthly
N	200	200	200	200	200	200	200	200	200	200	200	200
R^2^	0.764	0.803	0.666	0.707	0.634	0.683	0.827	0.838	0.862	0.861	0.585	0.680

The table provides the results from the categories selected to best fit the subjective well-being data using a stepwise regression. Observations are weekly at the national level. Robust standard errors in parentheses

*** p<0.01

** p<0.05

* p<0.1.Use of a Newey-West estimator to account for autocorrelation does not substantially change results. Dependent and independent variables are standardized within the training sample.

**Table 2:** Regression of categories on subjective well-being data–negative affects (national level).

	Anger		Stress		Worry		Sadness	
	(13)	(14)	(15)	(16)	(17)	(18)	(19)	(20)
**Material conditions**								
Job Search	0.315***	0.571***	0.309***	0.422***	0.725***	0.786***	0.479***	0.564***
	(0.114)	(0.067)	(0.050)	(0.051)	(0.042)	(0.044)	(0.040)	(0.060)
Job Market							-0.445***	-0.518***
							(0.124)	(0.099)
Financial Security	1.012***	1.234***	0.325***	0.401***	0.295***	0.450***	0.240***	0.551***
	(0.170)	(0.180)	(0.076)	(0.076)	(0.091)	(0.094)	(0.063)	(0.111)
**Social**								
Family Life	0.514***	0.416***	0.539***	0.470***			-0.447***	-0.418***
	(0.098)	(0.082)	(0.047)	(0.048)			(0.043)	(0.063)
Family Stress					0.217***	0.165**		
					(0.081)	(0.080)		
Civic Engagement			-0.271***	-0.215**				
			(0.080)	(0.086)				
Personal Security				-0.073**	0.078**		0.315***	0.258***
				(0.036)	(0.039)		(0.049)	(0.051)
**Health and Wellness**								
Healthy Habits	-0.326**		-0.435***	-0.339***	-0.362***	-0.312***	-0.172**	
	(0.143)		(0.059)	(0.057)	(0.050)	(0.046)	(0.066)	
Summer Leisure	-0.343**	-1.521***	-0.492***	-0.860***	-0.745***	-0.863***	-0.282***	-0.783***
	(0.160)	(0.308)	(0.076)	(0.142)	(0.080)	(0.136)	(0.071)	(0.180)
Health Conditions	-0.475***		0.193**	0.278**	-0.200***	-0.342***	-0.450***	-0.375***
	(0.142)		(0.077)	(0.113)	(0.072)	(0.086)	(0.116)	(0.130)
Education and Ideals		-1.092***		-0.367***			0.486***	
		(0.182)		(0.136)			(0.151)	
Time dummies	Quarterly	Monthly	Quarterly	Monthly	Quarterly	Monthly	Quarterly	Monthly
N	200	200	200	200	200	200	200	200
R^2^	0.527	0.606	0.884	0.906	0.862	0.880	0.783	0.822

The table provides the results from the categories selected to best fit the subjective well-being data using a stepwise regression. Observations are weekly at the national level from 2009–2012. Robust standard errors in parentheses

*** p<0.01

** p<0.05

* p<0.1.Use of a Newey-West estimator to account for autocorrelation does not substantially change results. Dependent and independent variables are standardized within the training sample.

Table 3 (Life Evaluation and Positive Affects) and Table 4 (Negative Affects) present the results for the state-level model, for all states together and also for red states (those where less than 45% of the vote went to Obama) and blue states separately. While there are some differences, there are important similarities: searches related to *Job Search* are consistently associated with higher negative affect, *Civic Engagement* is consistently associated with higher life evaluation, and *Healthy habits* is consistently associated with higher positive affect and lower negative affect. While there are some differences between the US-level and state-level models, several categories are consistently important to predicting different facets of well-being across all models.

**Table 3:** Regression of categories on subjective well-being data–life evaluation and positive affects (state level).

	Life Evaluation Today			Life Evaluation in 5 years			Happiness			Laugh			Learn			Respect		
US States:	ALL	BLUE	RED	ALL	BLUE	RED	ALL	BLUE	RED	ALL	BLUE	RED	ALL	BLUE	RED	ALL	BLUE	RED
**Material Conditions**																		
Job Search	-0.199***		-0.253***	-0.103***		-0.168***	-0.139**		-0.192***									-0.223***
	(0.056)		(0.067)	(0.022)		(0.049)	(0.061)		(0.049)									(0.076)
Job Market		-0.807***					-0.169**	-0.562***			-0.347***		-0.118**	-0.591***				
		(0.122)					(0.069)	(0.102)			(0.061)		(0.056)	(0.149)				
Financial Security					-0.064**	0.318***												0.172**
					(0.027)	(0.069)												(0.078)
**Social**																		
Family Life		0.225***	-0.246**	0.107***	0.184***					0.262***	0.418***		0.209***	0.222***				
		(0.066)	(0.106)	(0.036)	(0.033)					(0.070)	(0.077)		(0.049)	(0.063)				
Civic Engagement	0.256***	0.174**	0.382***											-0.197***				
	(0.075)	(0.075)	(0.092)											(0.061)				
Personal Security		0.167**	-0.246**										0.182**	0.375***				
		(0.073)	(0.106)										(0.070)	(0.097)				
**Health and Wellness**																		
Healthy Habits	0.239***						0.325***	0.395***	0.335***	0.216**		0.449***	0.198***	0.240***	0.219***			
	(0.083)						(0.068)	(0.064)	(0.086)	(0.097)		(0.084)	(0.060)	(0.078)	(0.064)			
Summer Leisure																		
Health Conditions		0.412***							-0.384***									
		(0.117)							(0.090)									
Education and Ideals	-0.321***	-0.807***				-0.256**	-0.248**	-0.562***		-0.478***	-0.347***	-0.562***	-0.472***	-0.591***	-0.346***			
	(0.096)	(0.122)				(0.114)	(0.123)	(0.102)		(0.076)	(0.061)	(0.074)	(0.089)	(0.149)	(0.065)			
Time dummies	Monthly			Monthly			Monthly			Monthly			Monthly			Monthly		
N	406	214	192	405	213	192	406	214	192	406	214	192	406	214	192	406	214	192
R^2^	0.686	0.757	0.620	0.821	0.863	0.798	0.706	0.629	0.753	0.613	0.531	0.678	0.805	0.766	0.831	0.402	0.364	0.484

The table provides the results from the categories selected to best fit the subjective well-being data using a stepwise regression. Observations are biannual at the state level (twice per year for 50 states from 2009–2012). Robust standard errors clustered at the state level in parentheses

*** p<0.01

** p<0.05

* p<0.1. Dependent and independent variables are standardized within the training sample.

**Table 4:** Regression of categories on subjective well-being data–negative affects (state level).

	Anger			Stress			Worry			Sadness		
US States:	ALL	BLUE	RED	ALL	BLUE	RED	ALL	BLUE	RED	ALL	BLUE	RED
**Material Conditions**												
Job Search	0.187***	0.185***	0.250***		0.114**		0.181***	0.204***	0.224***	0.221***	0.238***	0.178***
	(0.041)	(0.063)	(0.076)		(0.047)		(0.048)	(0.056)	(0.039)	(0.037)	(0.061)	(0.057)
Job Market								0.301**		0.147**	0.361***	0.214***
								(0.117)		(0.059)	(0.119)	(0.071)
Financial Security												
**Social**												
Family Life												
Civic Engagement			-0.325**	-0.104***	-0.199***	-0.101**						
			(0.133)	(0.030)	(0.054)	(0.040)						
Personal Security												
**Health and Wellness**												
Healthy Habits							-0.266***	-0.311***	-0.115***	-0.159***	-0.192**	-0.188**
							(0.079)	(0.101)	(0.036)	(0.054)	(0.086)	(0.071)
Summer Leisure	-0.096**						-0.109**	-0.185***			-0.251**	
	(0.048)						(0.050)	(0.064)			(0.092)	
Health Conditions		-0.115**	0.227**									
		(0.054)	(0.095)									
Education and Ideals							0.237***	0.301**			0.361***	
							(0.087)	(0.117)			(0.119)	
Time dummies	Monthly			Monthly			Monthly			Monthly		
N	406	214	192	406	214	192	406	214	192	406	214	192
R^2^	0.561	0.511	0.627	0.688	0.673	0.710	0.729	0.741	0.719	0.739	0.707	0.782

The table provides the results from the categories selected to best fit the subjective well-being data using a stepwise regression. Observations are biannual at the state level (twice per year for 50 states from 2009–2012). Robust standard errors clustered at the state level in parentheses

*** p<0.01

** p<0.05

* p<0.1. Dependent and independent variables are standardized within the training sample.

**Fig 1:**
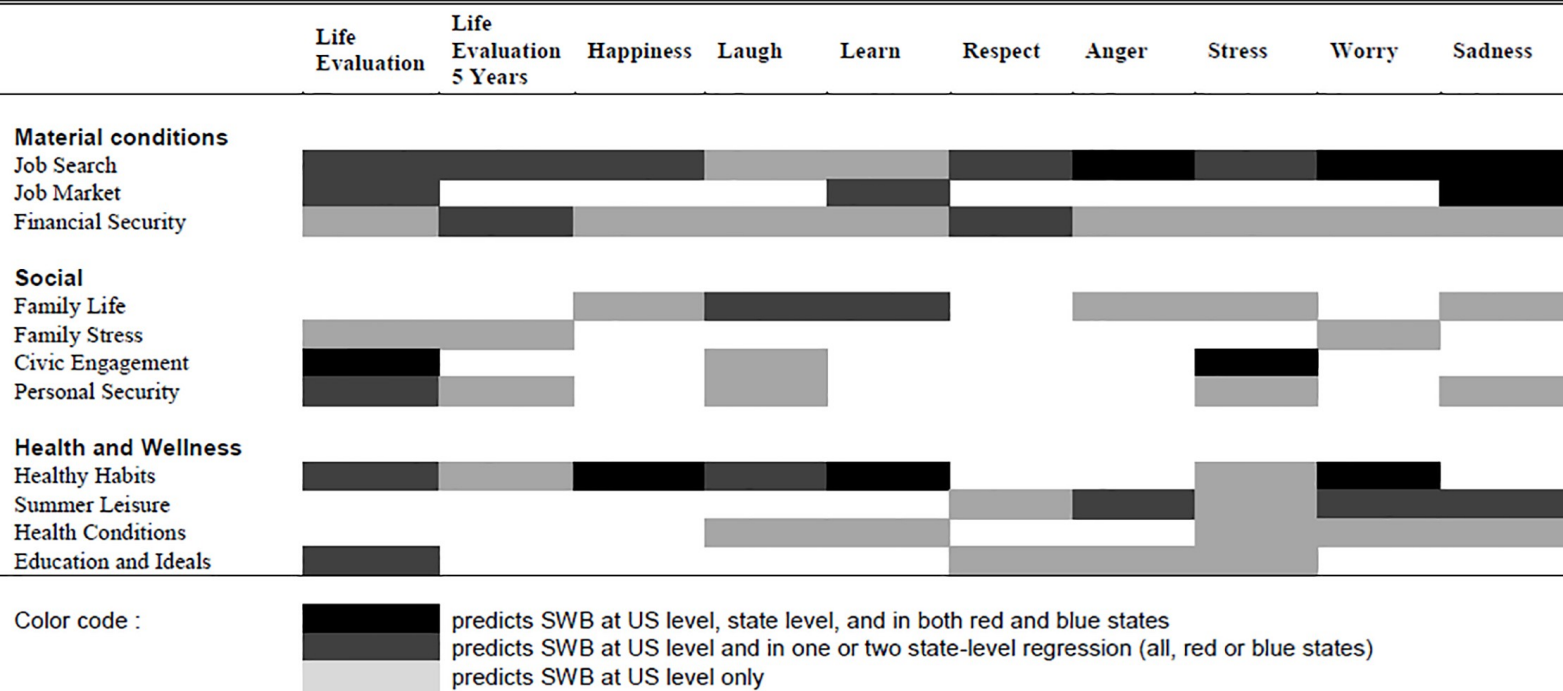
Mapping of significant predictors of subjective well-being across models. The figure shows the significance of different predictors across models. Job search, job market, civic engagement and healthy habits are all consistently important to predicting well-being across models.

Fig 1 provides a mapping of the significance of the different life dimensions: *Job Search*, *Civic Engagement*, and *Healthy Habits* seem to be particularly important predictors of well-being across models and samples.

Table 5 reports the results of a variance decomposition exercise from the US-level model. Overall, it appears that, for all subjective well-being variables except stress, material conditions are the most important family of predictors, followed by social factors and health/wellness categories. At the category level, the most important variables are job search, financial security, summer leisure and family life. Regarding stress, the variable appears to be mostly explained by family life, summer leisure and healthy habits.

**Table 5:** Decomposition of the explained variance of subjective well-being variables.

	Life Evaluation	Life Evaluation 5 Years	Happiness	Laugh	Learn	Respect	Anger	Stress	Worry	Sadness	Average
	(1)	(2)	(3)	(4)	(5)	(6)	(7)	(8)	(9)	(10)	(11)
**Material conditions**	**0.601**	**0.510**	**0.390**	**0.438**	**0.486**	**0.261**	**0.649**	**-0.017**	**0.670**	**0.426**	**0.503**
Job Search	0.408	0.067	0.131	0.219	0.182	0.122	0.274	0.118	0.533	0.168	0.228
Job Market	0.142				0.032	0.096				0.115	0.090
Financial Security	0.051	0.442	0.259	0.220	0.271	0.043	0.376	-0.134	0.137	0.143	0.185
**Social**	**0.099**	**-0.031**	**0.073**	**0.365**	**0.249**	**0.000**	**0.061**	**0.342**	**0.061**	**0.276**	**0.278**
Family Life			0.073	0.074	0.249		0.061	0.325		0.146	0.156
Family Stress	0.066	-0.183							0.061		-0.019
Civic Engagement	0.039			0.149				0.016			0.068
Personal Security	-0.005	0.152		0.142				0.000		0.130	0.072
**Health and Wellness**	**0.085**	**0.119**	**-0.069**	**-0.235**	**-0.068**	**0.178**	**-0.422**	**0.396**	**0.111**	**0.224**	**0.008**
Healthy Habits	0.063	0.119	-0.069	-0.092	-0.046			0.135	0.036		0.021
Summer Leisure						0.137	-0.252	0.344	0.221	0.248	0.112
Health Conditions				-0.143	-0.022			0.061	-0.145	-0.024	-0.062
Education and Ideals	0.022					0.040	-0.170	-0.145			-0.063
**Contribution of time dummies**	**0.017**	**0.110**	**0.289**	**0.270**	**0.194**	**0.241**	**0.318**	**0.185**	**0.037**	**-0.104**	**0.185**
**R**^**2**^	**0.803**	**0.707**	**0.683**	**0.838**	**0.861**	**0.680**	**0.606**	**0.906**	**0.880**	**0.822**	**0.974**

The table shows the decomposition of the explained variance of subjective well-being based on a model that fits categories of internet search volume data to subjective well-being search data. Overall, it appears that, for all subjective well-being variables except stress, material conditions are the most important family of predictors, followed by social factors and health/wellness categories.

### Estimating subjective well-being in the United States and reliability of the model

Both US-level and state-level models are reliable in out of sample tests, they yield fairly consistent predictions when controlling for seasonal trends at the quarterly or at the monthly frequencies, and they perform better than a ‘benchmark model’ that uses only seasonal predictors. The procedural framework for developing the estimation is consistent at the state and national levels.

**Fig 2:**
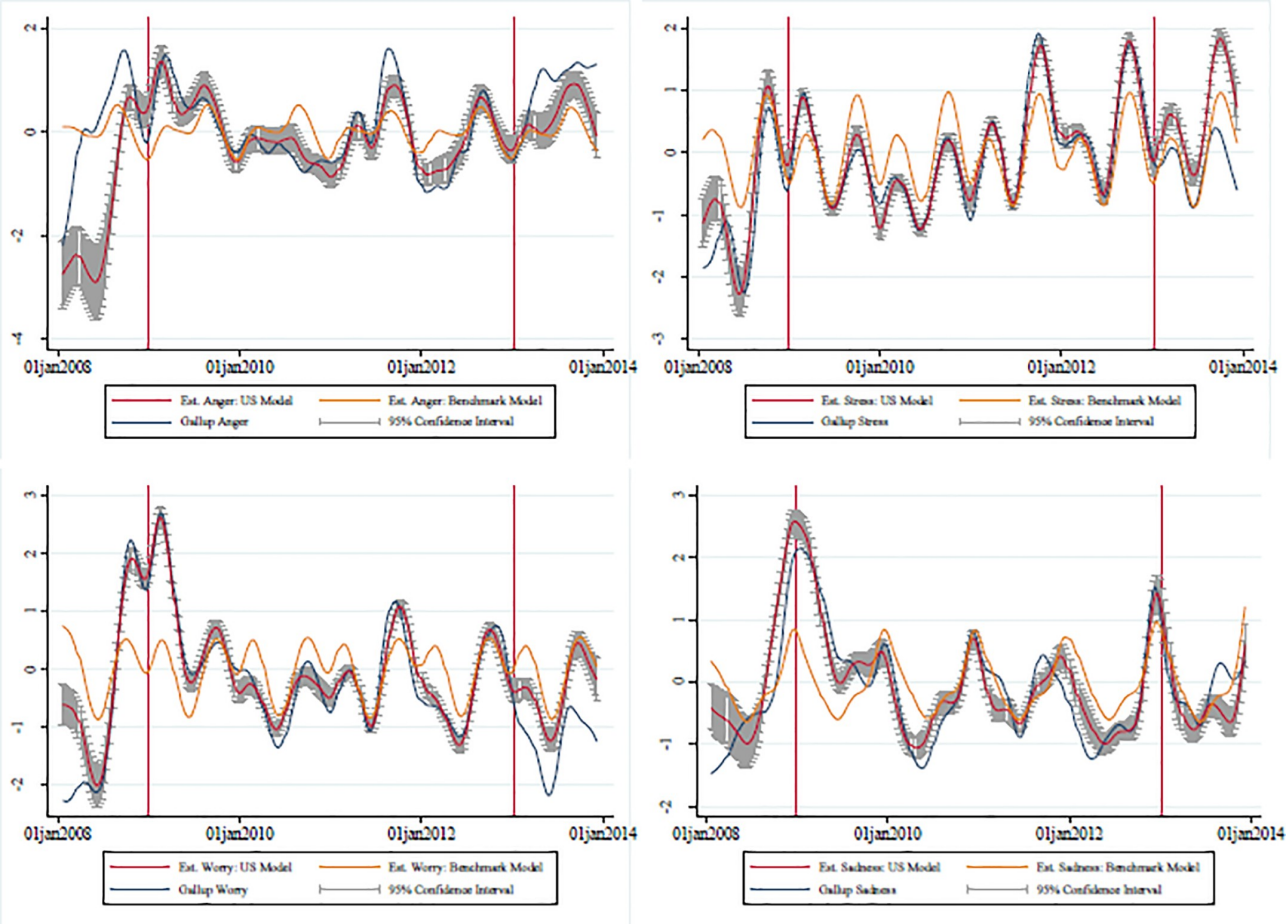
Predicted and observed subjective well-being (united states aggregate level). Graphs show the estimates (with confidence intervals) for subjective well-being at the US level, constructed using the US-model, in red, alongside estimates from the benchmark (seasonality only) model in yellow and the Gallup series in blue. Confidence intervals are constructed using 1000 draws. Training data is inside the red lines, and Testing data is outside the red lines. Correlations are given in Table 6.

Table 6 displays the correlations between the estimated and actual values of subjective well-being variables for both the US-level and state-level models as well as for the benchmark model used for comparison, over both the training and the test sub-periods. The US-level model uses categories and monthly dummies at the aggregate US level with one observation per week as presented in Tables 1 and 2; the state-level model uses categories and biannual data with fifty observations per biannual period and is presented in Tables 4 and 5; the benchmark model is also estimated at the US level and uses only monthly dummies. Fig 2 depicts the US-level estimated and observed subjective well-being variables and the 95% confidence interval for the estimates.

**Table 6:** Correlation between predicted and observed subjective well-being values.

	Life Evaluation	Life Evaluation 5 Years	Happiness	Laugh	Learn	Respect	Anger	Stress	Worry	Sadness	Average
Sample	(1)	(2)	(3)	(4)	(5)	(6)	(7)	(8)	(9)	(10)	
**US-level model**											
Training	0.93	0.92	0.88	0.97	0.96	0.91	0.91	0.97	0.97	0.94	0.936
Test 2008	0.84	0.76	0.80	0.88	0.47	0.13	0.6	0.92	0.95	0.90	0.725
Test 2013	0.94	0.84	0.59	0.82	0.55	0.77	0.61	0.79	0.86	0.66	0.743
**Benchmark model**											
Training	0.25	0.28	0.41	0.46	0.54	0.38	0.42	0.72	0.54	0.53	0.453
Test 2008	0.28	0.64	-0.10	0.15	0.87	0.48	0.58	0.79	0.45	0.59	0.473
Test 2013	0.85	0.66	0.15	0.66	0.17	0.58	0.47	0.87	0.81	0.71	0.593
**State-level model**											
Training	0.93	0.97	0.96	0.94	0.97	0.85	0.93	0.93	0.96	0.95	0.939
Test 2008	0.81	0.88	0.79	0.63	0.90	0.35	0.81	0.8	0.87	0.85	0.769
Test 2013	0.72	0.82	0.83	0.80	0.89	0.70	0.73	0.74	0.82	0.84	0.789

The table shows the correlation between the predicted series and the Gallup series (the blue lines on Fig 1) of ten different measures of subjective well-being (both smoothed). For the US-level model (the regression given in Tables 1 and 2 and the red lines with confidence intervals in Fig 1), the unit of observation is the week, and the training sample is the 200 week period that was used to fit the model, corresponding to the zones inside the red lines in Fig 1.The out of sample tests are the Test 2008 and Test 2013 rows, where the Test 2008 sample is the 50 weeks in 2008 that were not used to fit the model, and the Test 2014 sample is the 50 weeks in 2014 that were not used to fit the model. These periods correspond to the zones outside the red lines in Fig 1.The Benchmark model uses the same sample and time periods as the US-level model, but uses only month dummies. This predicted series is shown as the yellow line in Fig 1. The State-level model uses the Gallup biannual state data, where the years 2009–2012 provide the training sample (the regression given in Tables 3 and 4) and 2008 and 2013 are the out of sample tests. Fig 2 shows the scatterplot of predicted well-being to well-being in the Gallup data

For the US-level model, during the training sub-period, correlations between the estimated and actual series are high, as expected, with an average of 0.94. The correlations remain high in out-of-sample testing periods for most subjective well-being variables at the national level, with an average of 0.72 in 2008 and 0.74 in 2013. Two of the ten affects stand out as being particularly difficult to estimate (out of sample): *Learn* (0.47 in 2008 and 0.55 in 2013) and *Respect* (-0.13 in 2008 but 0.77 in 2013). One possible reason for this is that these affects are not well defined or understood.

**Fig 3:**
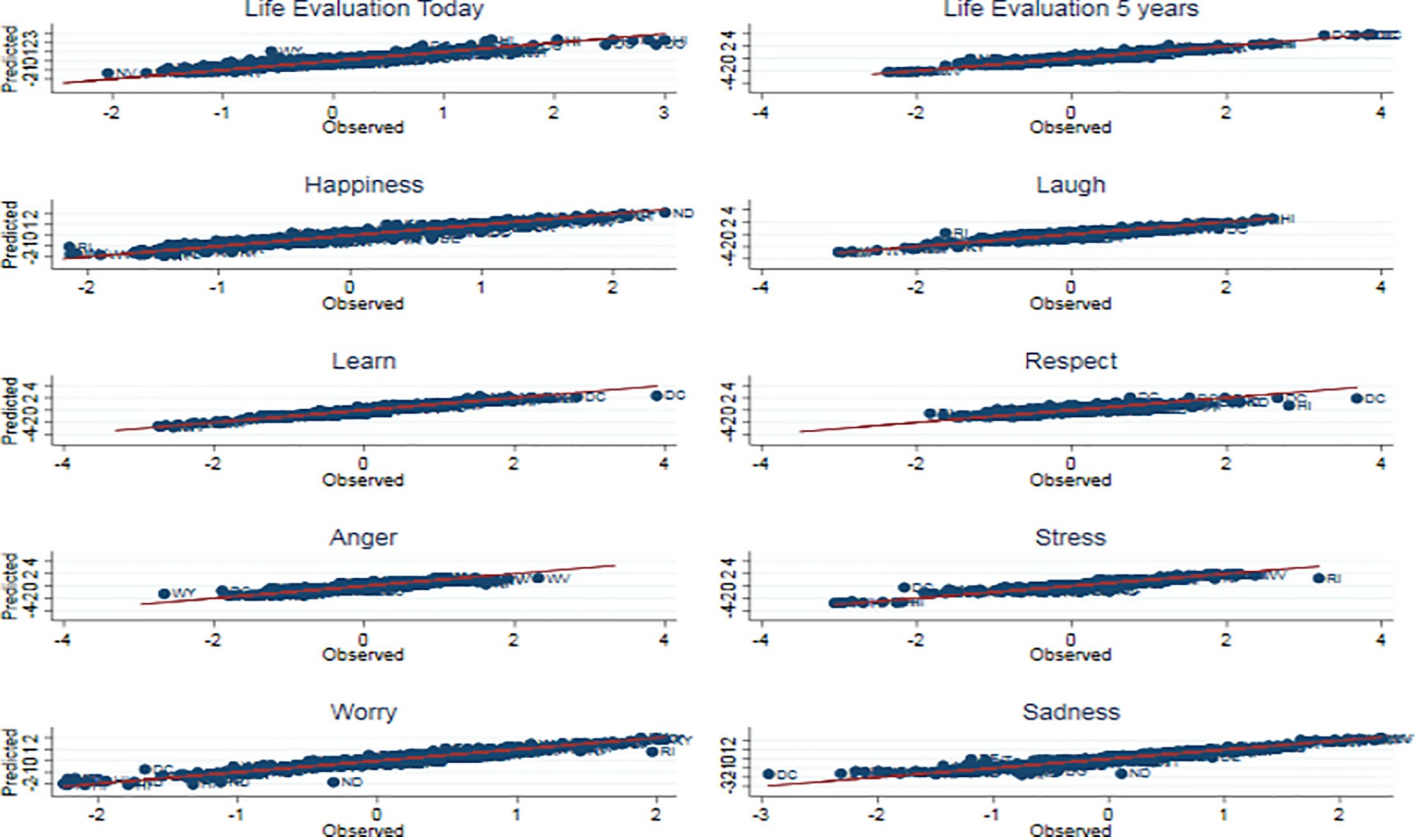
Biannual predicted subjective well-being and biannual Gallup data at state level, training and test samples (2008–2013). The figure shows a scatterplot of the predicted life evaluation today (y-axis) and Gallup surveyed life evaluation today (x-axis) at the biannual state level, both series normalized with mean 0 and standard deviation 1. Correlations are given in Table 6.

The state-level model yields estimates with similarly high correlations with actual data in and out of sample, on average, and Fig 3 shows the scatterplots of the estimated and observed state-level biannual subjective well-being indicators. The US-level model and the state-level models both perform much better than the benchmark model, indicating that the Google Trends data add information to the seasonality observed in the series. The benchmark model performs relatively poorly even in the training set, with an average correlation of 0.45.

The objective of this exercise is to obtain a combination of words that is able to estimate the evolution -and not the level- of subjective well-being. Note that a high correlation does not necessarily imply an accurate estimate, as the correlation measures the degree to which the two data move together, rather than whether they are equal. For example, the 2008 test period has a very high correlation for *Life evaluation*, but visual inspection of the graph shows that while the series move together, the estimated series is much higher. In the case of *Life evaluation* this may be due to the change in the ordering of questions in the Gallup survey that took place at about this time, and is thought to depress the overall *Life evaluation* measure in 2008, as reported by Deaton [27].

## Discussion

This paper constructs robust predictors of subjective well-being variables in the United States while drawing from a very large amount of search engine data covering the period of 2008–2013. The choice of the initial set of keywords is grounded in theory and in the existing literature on the empirical determinants of subjective well-being. Among this initial set, keywords are selected and grouped together to form composite categories when they pass two statistical tests, namely the absence of a strong deterministic time trend and the joint consistency of keywords grouped into categories. Out of 845 initial keywords, 215 pass the selection tests. The resulting composite categories help filter the relevant information out of a large number of noisy measures, which is often an important concern when working with internet search data and Big Data more generally. As a result, the model successfully estimates the out-of-sample evolution of most subjective well-being measures at a one-year horizon. Regarding future research, this paper lays the groundwork for constructing well-being indices at the local level (state or metropolitan area), which might then be used to measure the impact of local shocks or policy reforms on well-being in the United States. Two use cases described below illustrate this possibility.

Overall, the coefficients in our models are in line with the literature on subjective well-being, which supports their validity. The consistent negative relationship of *Job Search* (which relates to searching for a job) is in line with the finding from other studies that underline the importance of employment as a foundation of subjective well-being: having a job is one of the strongest correlates of life satisfaction and happiness, while, conversely, being unemployed is highly detrimental to life satisfaction, notwithstanding the loss of income that this entails [28], and is most difficult to adapt to [29]. In our study, some keywords in the category *Job Search* are strongly related to searching for a job from unemployment or non-employment (e.g. ‘unemployment benefits’, ‘job fair’, ‘apprenticeships’), while some others address layoffs (‘layoffs’, ‘severance pay’) or could come from employed workers in search of another job (e.g. ‘part time job’, ‘career fair’). As a result, the category *Job Search* seeks to elicit job concerns across a range of individual situations. *Civic Engagement* is related to the importance of social capital, which has been amply demonstrated to be strongly associated with subjective well-being [30,31]. *Healthy Habits*, notably physical exercise, are associated with less depression and anxiety and improved mood [32]. Family, health and security are identified, through choices, as extremely important in terms of people’s happiness by Benjamin et al. [33] and these categories coincide with the “satisfaction domains” that have been explored by the Leyden school [34]. Many of these patterns of subjective well-being are summarized in The World Happiness Report [2].

Two categories have inconsistent signs. *Family Life* is associated with more Happiness and Laughter, and less Sadness, but also with more Anger and Stress. The finding of inconsistent associations of *Family Life* may reflect the complicated nature of interactions with children and is in fact consistent with the finding in Deaton and Stone [6] that parents experience both more daily joy and more daily stress than non-parents [35]. *Personal Security*, which includes keywords such as ‘violent crime’, ‘assault’, ‘murder’ and ‘crime rate’, is, as expected, negatively associated with Life Evaluation in 5 years and Laughter, and positively associated with Sadness (generally, living in a high crime area is associated with lower subjective well-being [36]). At the same time, *Personal Security* appears to be positively associated with Life Evaluation Today, which is potentially a result of multicollinearity among the predictors, as suggested by the negative explained variance in Table 5 (itself driven by strong negative covariance between *Personal Security* and other explanatory variables). In any case, the latter explained variance is also very small, so that this category displays very weak explanatory power with respect to Life Evaluation Today.

### Two examples of use cases

We provide two examples of the way fluctuations in estimated well-being at the state level can be used to better understand the relationship of well-being to events. The first example is the shift in estimated well-being in red states (defined as those where fewer than 45% voted for Obama) following Obama’s election. Table 7 provides these estimates, with well-being in red states dropping (relative to non-red states) following Obama’s election in the expected direction in 8 out of 10 cases. Two composite categories explain the estimated decrease in positive well-being and the increase in negative well-being among red states: the rise of searches for words associated with job search and with rights, moral issues and education.

**Table 7:** Well-being in red states following election of Barack Obama.

	Life Evaluation	Life Evaluation 5 Years	Happiness	Laugh	Learn	Respect	Anger	Stress	Worry	Sadness
	(1)	(2)	(3)	(4)	(5)	(6)	(7)	(8)	(9)	(10)
										
Red state x post-election	-0.0940*	-0.141***	-0.0529*	-0.0560**	-0.0301	-0.0603***	0.0333*	-0.0525**	0.0659**	0.0809**
	(0.0477)	(0.0388)	(0.0270)	(0.0221)	(0.0196)	(0.0225)	(0.0191)	(0.0211)	(0.0315)	(0.0305)
Post-election	0.467***	0.465***	-0.342***	-0.204***	0.203***	0.177***	0.275***	0.186***	0.510***	0.428***
	(0.0285)	(0.0262)	(0.0185)	(0.0136)	(0.0124)	(0.0120)	(0.0112)	(0.0131)	(0.0193)	(0.0205)
Red state	-0.0333	0.0194	-0.00177	0.0122	-0.0137	-0.0144	0.0102	0.0286**	0.0117	-0.0396*
	(0.0448)	(0.0316)	(0.0213)	(0.0163)	(0.0173)	(0.0228)	(0.0163)	(0.0107)	(0.0253)	(0.0204)
Month dummies	Yes	Yes	Yes	Yes	Yes	Yes	Yes	Yes	Yes	Yes
State dummies	Yes	Yes	Yes	Yes	Yes	Yes	Yes	Yes	Yes	Yes
Observations	1,071	1,071	1,071	1,071	1,071	1,071	1,071	1,071	1,071	1,071
R-squared	0.943	0.969	0.967	0.972	0.980	0.988	0.973	0.985	0.946	0.971
**Structural explanations**										
***Job search***										
Significant change in red states following election	**+**	**+**	**+**	**+**	**+**	**+**	**+**	**+**	**+**	**+**
Elasticity in state-level model	**-**	**-**	**-**	0	0	0	**+**	0	**+**	**+**
Expected election effect	**-**	**-**	**-**	0	0	0	**+**	0	**+**	**+**
***Financial security***										
Significant change in red states following election	**-**	**-**	**-**	**-**	**-**	**-**	**-**	**-**	**-**	**-**
Elasticity in state-level model	0	0	0	0	0	0	0	0	0	0
Expected election effect	0	0	0	0	0	0	0	0	0	0
***Personal security***										
Significant change in red states following election	**+**	**+**	**+**	**+**	**+**	**+**	**+**	**+**	**+**	**+**
Elasticity in state-level model	0	0	0	0	**+**	0	0	0	0	0
Expected election effect	0	0	0	0	**+**	0	0	0	0	0
***Summer leisure***										
Significant change in red states following election	+	+	+	+	**+**	**+**	**+**	**+**	**+**	**+**
Elasticity in state-level model	0	0	0	0	0	0	**-**	0	**-**	0
Expected treatment effect	0	0	0	0	0	0	**-**	0	**-**	0
***Education and ideals***										
Significant change in red states following election	**+**	**+**	**+**	**+**	**+**	**+**	**+**	**+**	**+**	**+**
Elasticity in state-level model	**-**	0	**-**	**-**	**-**	0	0	0	**+**	0
Expected election effect	**-**	0	**-**	**-**	**-**	0	0	0	**+**	0

The upper half of the table provides the difference in difference estimates for well-being in red states (defined as having less than 45% of the vote for Obama) and non-red states before and after the election of Obama in 2008. Robust standard errors are clustered at the state level and in parentheses. Due to the inclusion of time and state dummies, the only coefficients of interest are those of the interaction variable Red state x post-election. The lower half of the table shows, for each of the composite categories for which there is a significant difference-in-difference estimate, the direction of the change, the elasticity in the state-level model (that is, whether a positive change in the composite category should lead to a positive, negative, or zero change in the well-being variable, and the expected effect of the election.

*** p<0.01

** p<0.05

* p<0.1.

**Fig 4:**
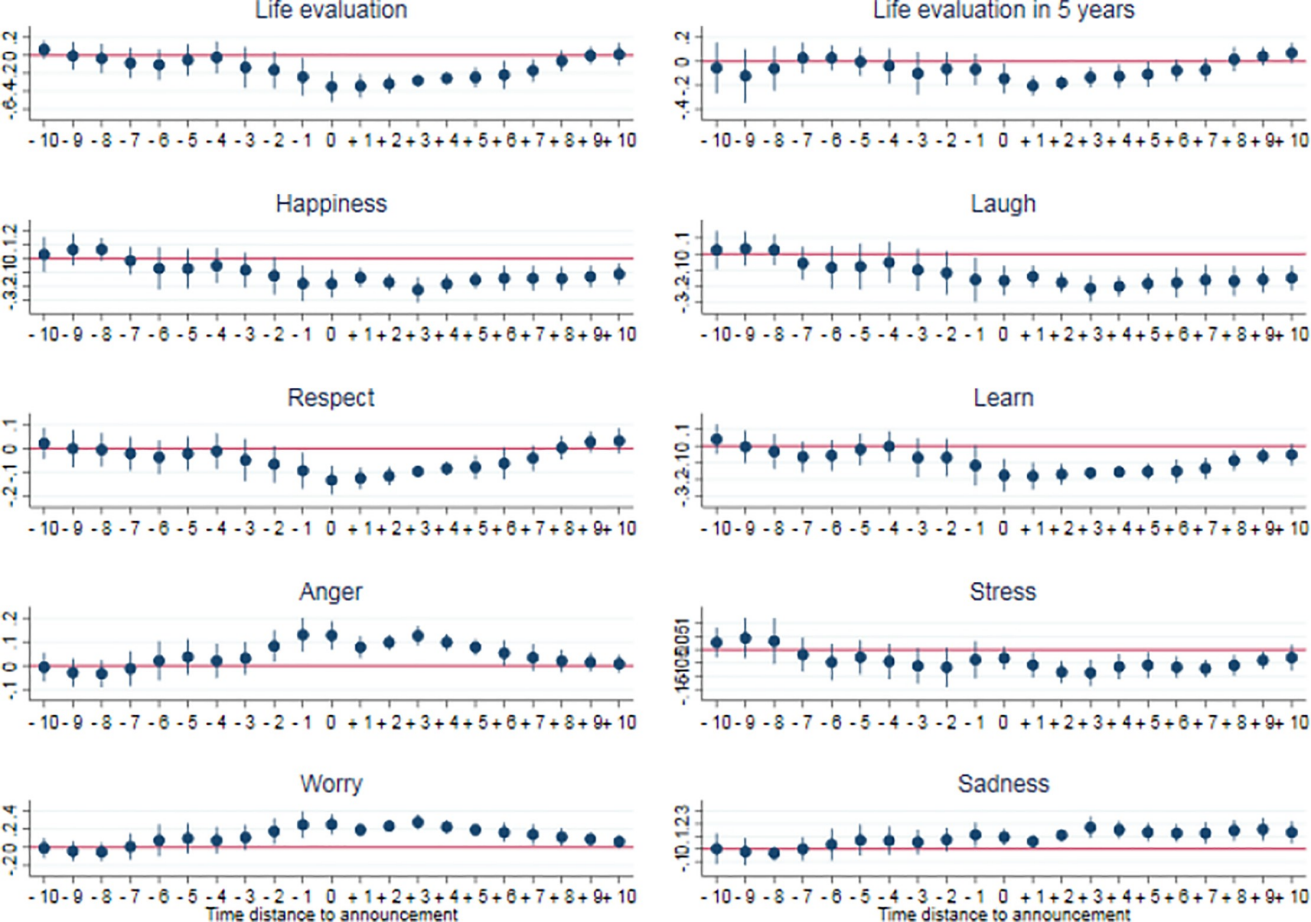
Well-being and mass layoff announcements. The figure shows the changes in estimated well-being in states where an announcement of a mass layoff (more than 1000 employees) has been made. The x-axis is the time around the announcement, where 0 is the month that the announcement is made, negative numbers indicate the number of months prior to the announcement, and positive numbers indicate the number of months after the announcement. The y-axis shows the difference, for that month, between states where an announcement has been made and other states.

The second example is the change in estimated well-being at the state level when an announcement that there will be mass layoffs is made. Fig 4 provides estimates for changes in subjective well-being in the states in the months surrounding notice of a mass layoff, both before and after the month of the notice of a mass layoff (where the zero on the x-axis represents the month in which the notice is given). At the time of the announcement, positive well-being generally declines and negative well-being generally increases, and then gradually recovers around six or seven months after notice has been given. The gradual recovery of well-being for these states is in line with the literature on subjective well-being and adaptation.

This paper is focused on “nowcasting” instead of “forecasting” because it is possible that the construction of the categories, and the model itself, may not be stable over time, and the factors that help us understand well-being in one period might not be perfectly applicable in another (see D’Amuri and Marcucci [14] for an example of using Google Trends data for forecasting, and Preis and Moat [37] for adaptative nowcasting). The period investigated here (2008–2013) included the economic crisis. During a later period, one might expect that other concerns become important predictors of well-being (for example, security concerns might loom larger in the years following 2013). Further work should expand the period under consideration, investigate the time-dependence of the model, and potentially implement a system for regular revision of the words used.

## Materials and methods

### Gallup data

As described previously in Algan et al. [38], the subjective well-being data is taken from Gallup Analytics, which is a daily telephone survey of at least 500 Americans aged 18 and older. The time span of the each of the ten series used in this paper covers 300 weeks from January 6, 2008 to January 4, 2014. More than 175,000 respondents are interviewed each year, and over 2 million interviews have been conducted to date since the start of the survey in 2008. The survey includes 6 measures of self-reported positive emotions (happiness, learn, life evaluation today and in 5 years, laugh, being respected) as well as 4 measures of negative emotions (anger, sadness, stress, worry). S1 Fig shows the evolution of the ten indicators over the period. The consequences of the Great Recession are visible on most subjective well-being indices: life evaluation today and in 5 years, *happiness* and *laugh* have dropped significantly in 2008–2009, while the percentages of people experiencing worry, anger, stress and sadness have increased at the same time. A second observation concerns the cyclicality of these variables, which all display large seasonal swings.

### Google search data

This paper uses data on the volume of internet searches for individual words, which are available from Google Trends (see Algan et al. [38]). The initial list of words is selected as follows. We extract two long lists of words potentially linked to subjective well-being outcomes. The first list comes from the Better Life Index Online Database, which records answers from data users to the question “What does a Better Life mean to you?” The second list is based on the American Time Use Survey, which records the daily activities undertaken by US citizens as well as the positive or negative emotions that are associated with these episodes. This selection method allows us to avoid a cherry picking of a limited set of search queries on Google. On the other hand, survey-based words may be disconnected from the day-to-day life of Americans if they do not include their usual internet queries or do not reflect their practical living conditions. As a consequence, we have added a set of words that were likely to be relevant to different life experiences related to subjective well-being, for example, job concerns (e.g. ‘unemployment’), poverty (‘coupons’) or family stress (‘women shelter’). In total, the initial database contains 827 words, of which 201 were related to material conditions (income, wealth, employment, and housing) 529 were related to quality of life (health, leisure, education, environment, civic life, personal security, subjective well-being, and social connectedness) and 97 were related to potentially taboo categories (pornography, hatred and racism, and conspiracy theories). The words that were used are likely to be highly specific to the United States, and potentially specific to the time period studied.

Data for each of the ten well-being variables from Gallup and the search volumes from Google Trends are available at different time intervals for the United States at the aggregate level, and we have used data at the weekly level in both cases, for the period 2008–2013. For each of the 300 weeks during the period, we have data on both search volume and surveyed well-being.

### State panel data

We have also obtained search volumes for the words from Google Trends for each of the 50 states, when available, at a monthly frequency. While Gallup data are collected from states, sample sizes are not large enough to produce high-frequency representative estimates. As a result, Gallup provides state-level data only at a biannual frequency, which consists of the aggregation of multiple waves from the high-frequency national sample. In some cases, the sample sizes for Gallup data are too low, and so we exclude all observations with sample size below 1500. We take six-month averages of the composite categories (discussed below) at the state level, and match this with the biannual state subjective well-being measures from Gallup to create a panel dataset with 597 observations.

### Challenges with the google trends data

As described previously in Algan et al. [38], the search volumes for individual words obtained from Google Trends pose several challenges for estimation. The Google Trends data on search volume is not the raw search volume; rather it is the proportion of total searches over a given period that included that keyword, normalized so that the highest volume over the period is equal to 100. This has several consequences: first, the value of the series obtained directly from Google Trends is difficult to interpret, as it depends not only on the volume of searches for a given word but also on the volume of other searches. Second, the value of the series on any given day cannot be compared between terms, since they are normalized to the maximum value by term. To deal with this issue, we normalize all search volumes so that they have a mean of zero and a standard deviation of one, since we are interested in how volume changes within a given term (rather than which terms have the highest search volumes overall).

There may be sharp spikes in the popularity of a given word. While some of these spikes are surely related to the degree to which the concept represented by this word is important in people’s lives, others are less directly related. The example of the spike in “divorce” searches induced by the divorce of Kim Kardashian (an American celebrity) from Kris Humphries in October 2010 is shown in S2 Fig. This is a concern for estimation as it creates a risk of over-fitting: if a sufficient number of words have a sufficient number of spikes, one could estimate almost any series perfectly (though with poor out of sample performance). Spikes also tend to create unstable specification selection (in that the inclusion of one term is highly dependent on the inclusion of another). We reduce this risk by smoothing the data using a five period (week) moving average and by creating composite category indicators to dampen the importance of a shock in any individual keyword. The results are not sensitive to the number of periods in the moving average.

Other words show “cliffs”, where volume is at or near zero for some substantial period (see S3 Fig), and it is difficult to know whether it is because volume was zero or because there is an issue with the way the Google trends data is compiled. These cliffs pose an issue similar to that of the spikes, especially since words have cliffs at different points (that is, it is not a uniform discontinuity). However, we do not wish to exclude all zeros, because some zeros reflect very low volume. To address this issue, we dropped any word with more than five zeros during the period (changing the number of allowable zeros does not substantially change the results). This results in a loss of information, as we have to exclude many terms that are potentially salient and important, such as mace spray (Stephens-Davidowitz [23] provides an algorithm to recover very low search volumes, so that they do not appear as zeros).

We observed an unexplained discontinuity in many series from the last week of December 2010, to the first week of January 2011. An example for the word “pregnancy” is provided in S4 Fig. We believe this discontinuity to be related to the change in the algorithm used by Google to localize the searches in January 2011. To adjust for this discontinuity, we calculate the average index in December and January for the unaffected years, take the average change during the unaffected years, subtract this unaffected average change from the observed change from December 2010 to January 2011, and adjust all data from 2011 onwards using this difference. That is, we assume that the change from December 2010 to January 2011 should be the same as in the other years, and we adjust accordingly. While we are undoubtedly losing some information with this adjustment, there should not be any bias introduced.

Many of the words have a strong time trend. The example given in S5 Fig is “teeth hurt”, where the time trend from 2008 to 2014 explains 89% of the variance in frequency. The consistent relative increase in the search volume of “pain” may be due to at least two possibilities: people are feeling more pain, or people are feeling the same amount of pain but are turning towards the internet for medical care as a general cultural shift. We would like to capture the first, but we have no way to distinguish it from the second. In this case we chose to drop all words where the R^2^ from a regression of time on the keyword is greater than 0.6, and to visually investigate words between 0.5 and 0.6. This process reduces the number of available words from 845 to 554. We may be losing some important information in this step, but we feel the danger posed by conflating shifts in the way the internet is used with how people are actually feeling is more severe.

Finally, many words exhibit extreme seasonality (particularly those that have to do with leisure). Since some of the subjective well-being variables also exhibit seasonality, this is a major concern, as words might be correlated with a given subjective well-being variable merely because they follow the same seasonal trend. We guard against this by using month dummies in all specification with one small modification: the months of December and January exhibit consistent and dramatic intra-month patterns, presumably due to the Christmas holidays and New Year’s Eve. We thus also construct additional dummy controls for the each of the four weeks of those two months (and so the December and January month dummies are dropped).

We note that both survey data and internet search data face problems of selection. Telephone survey respondents, for example, are likely to be older than the population in general. Weights are used to compensate for selection effects but are unlikely to do so perfectly, as some dimensions of selection into response are likely unobserved. Internet search data, on the other hand, is likely to be skewed in the other direction, towards younger people who are more frequent internet users.

### Mass layoff data

Federal and state Worker Adjustment and Retraining Notice (WARN) Acts require businesses above a certain size to give advance notice of layoffs over a certain threshold of number of workers (the threshold depends on the state, but any business anticipating a layoff of 1000 people would be required to give notice). We obtained data on large-scale layoff notices (over 1000 employees) from 2008–2013 for sixteen states (those which made data on WARN notices downloadable for this period).

The data that support the findings of this study are available from Gallup but restrictions apply to their availability as access to these data was purchased for the current study. The raw data from Google Trends is freely available for download, however the terms of use prohibit distribution of these data without permission. We have complied with both Gallup and Google Trend API terms of use and services. The code used to construct the datasets and carry out the analysis is public and can be downloaded from the Open Science Framework (https://osf.io/).

### Formation of categories

We combine individual words into composite categories that are used as predictors of subjective well-being, which, in addition to reducing the number of potential predictors, has the added advantage of limiting the noise due to any individual variable. This allows for the possibility of continuous and ongoing estimation of subjective well-being, as it allows any word that may become unusable in the future due to internet ‘cascades’ or cultural change to be removed without greatly altering the significance of its category as a predictor of subjective well-being. In addition, constructing categories offers more visibility on the nature of correlates of subjective well-being variables, and allows disentangling the aspects of life (e.g. housing, employment, health, leisure…) that correlate most with different types of subjective well-being variables, such as short-run emotional affects (e.g. feelings of happiness, stress and worry) and cognitive variables such as life evaluation.

The grouping of words into categories must be coherent both logically and statistically. The words grouped together must meet a common sense test, and they must also pass a statistical test, which implies first conducting factor analysis (using only the training data) and then calculating the Cronbach’s alpha, which measures the cross-correlation of the components and is an estimate of the internal consistency and reliability of the constructed category. As many words exhibit seasonality, and different words may exhibit similar patterns of seasonality without sharing the same meaning, we used the residuals of a regression over month and week dummies (to remove seasonal effects) in order to test the coherence of the word grouping. However, we used the raw data (without the removal of seasonal variations) in order to construct the categories.

The grouping took place at the national level using the national dataset. We grouped the words into categories (such as jobs or family), then ran a factor analysis within each category. Words were excluded if the factor loading was negative or less than 0.3, and many words were not used because they did not fit consistently with any category grouping (in a handful of cases terms with slightly lower factor loadings were retained because the correlation with other words in the group was high for most of the period but they did not share one shock, such as home alarm and mugging which share most patterns with other words in the Personal Security category but do not share the shock of the Sandy Hook murders). We use 215 words of the 554 words available after cleaning. We only used words with a positive factor loading. The same grouping was used at the national and state level.

Categories themselves are constructed on the basis of a simple average of the z-scores for both the national and state level. This is to avoid the structure of a category from depending on the inclusion of a single word, and to facilitate future construction and revision of the categories, in case one of the components needs to be dropped due to an unexpected peak. Using an estimate of a latent variable calculated from the factor loadings produces substantially similar results. Due to space constraints, the factor loadings for each of the categories as well as the results using the estimated latent variables instead of z-score averages are available from the authors upon request.

Words were grouped into twelve domains that can be organized into three aspects of life: Material Conditions (*Job Search*, *Job Market*, *Financial Security* and *Home Finance*), Social (*Family Stress*, *Family Time*, *Civic Engagement* and *Personal Security*), and Health and Wellness (Healthy Habits, Health Conditions, Summer Activities and Education and Ideals). We intentionally exclude *Home Finance* from the model, due to the predominance of words critically linked to the financial crisis (“mortgage”, for example) during this period, making the importance of these words in predicting subjective well-being highly time-specific. Note that the words in *Job Market* and *Job Search* do not group together, and the types of words in each category give some intuition as to why: *Job Search* seems to be related to searching for a job (any job) from unemployment, while *Job Market* seems to be related to job quality, which might reflect searching in a looser job market. The lowest Cronbach’s alpha (for *Healthy Habits*) is 0.84, which is still reassuringly high. A commonly accepted rule of thumb sets 0.7 as a threshold for an acceptable degree of internal consistency [39,40]. S1 Table provides the composite categories and their components.

S6 Fig shows the evolution of the category variables over time, and S7 Fig provides some comparison of those trends to other social trends reflected in administrative data. *Job Search* and *Job Market* both show the severity of the crisis in 2008–2009 and the subsequent improvement of labor market conditions. Several of the categories exhibit sharp seasonal trends, with dips or jumps around the holidays. Note that *Job Search* peaks in 2009, when the unemployment rate was increasing the most quickly, and *Job Market* peaks in early 2010, when the unemployment rate was stabilizing and starting to drop, and *Job Search* shows less of a seasonal drop around Christmas than *Job Market*. Similarly, the declining trend in *Financial Security* and *Home Finance* seem to indicate that Americans have been less and less preoccupied by housing conditions and their financial conditions over the period. *Financial Security* also closely tracks bankruptcy (Chapter 11) petitions in US courts. Personal *Security* shows a slow decrease from 2009 to 2012 but a marked jump around December 2012 –one possibility is that this jump shows the fears and grief of the public following the Sandy Hook Elementary School shooting on December 14, 2012. *Family Life* shows an increasing trend over the period, whereas *Family Stress* decreases after the financial crisis, and the decrease in *Family Stress* maps onto the decrease in Intimate Crime incidents reported by the FBI. *Civic Engagement* is somewhat higher during the financial crisis but not markedly so, as are *Health Problems* and *Education and Ideals*. *Healthy Habits* showed a rebound as the economy began to recover; its sharp discontinuities every January are remarkable and may reflect New Year’s Eve resolutions. Finally, *Summer Leisure* exhibits a slight downward trend with a high seasonality, and the smoothed and seasonally adjusted series maps onto consumer spending on entertainment.

### Test and training periods

We then build a model using these categories to estimate well-being, training the model on the Gallup data, using either aggregate US data at the weekly level or the panel state data at the biannual level. Regarding model selection and out-of-sample estimation, we divide the sample into a “training” and “test” sample, and use the training sample to build the model, and evaluate its performance on the test sample. Data from the beginning of 2009 through the end of 2012 is used for the training set (200 weeks in the US data and 406 observations in the state dataset), while data from 2008 is used for one test set and data from 2013 for the other (a total of 100 weeks in the US dataset and about 100 observation for each year for the state dataset). The test sample brackets the training data, but does not overlap. The reason for the symmetrical test sets is that we would like to construct an index that estimates as well for periods of crises (i.e. the 2008 economic crisis) as for periods of relative stability.

### Selection of predictor categories

Our goal is to build a model that estimates the evolution of well-being using data from Google Trends. Such a model must avoid overfitting and underfitting the training data. Overfitting (as would be the case if all available individual words and month dummies were used) would result in high explanatory power in sample, with R^2^ statistics near one, but low predictive power out of sample, because the model would be calibrated to fit random variations in the sample rather than actual relationships. Conversely, using too few predictors creates a risk of underfitting of the dependent variable, which also would yield poor estimates. This dual problem is pervasive in the world of ‘Big Data’, which is often characterized by the availability of a lot of information (i.e., in our setting, a large number of potential explanatory variables) and substantial noise (each variable being a poor predictor of the dependent variable). Any selection procedure can be distorted when there are many redundant or highly correlated covariates in the sense that the detection of robust and distinct predictors gets diluted away from covariates that are not highly correlated with other predictors. This well-known problem is related to the independence of irrelevant alternatives problem in discrete choice models [41].

Part of this problem is addressed by reducing the number of possible explanatory variables by grouping words into the composites (discussed above) that are more stable and can be interpreted with more confidence than individual words. This procedure leaves 12 potential explanatory variables from which the model components must be selected. We use a backward stepwise procedure for variable selection; results obtained from stepwise selection either perform better than or do not differ greatly from alternative selection methods, including Bayesian Model Averaging, manual deletion of non-significant terms, excluding month dummies or including quarterly dummies, or even including all possible predictors, in addition to the Newey-West estimator. Using the selected variables, we calculate a model based on a simple OLS regression with robust standard errors at the level of the United States. Finally, we use the weights from the model (estimated over the training period) to estimate subjective well-being over the whole period, while the prediction performance is reported separately over training and test sub-periods (see Table 6). To demonstrate that the selected model is not a result of overfitting aggravated by the inclusion of monthly dummies, we carry out this procedure using monthly and quarterly dummies and show that the results are largely similar (Tables 1 and 2).

We repeat this exercise at the state level using the state level panel dataset at the biannual frequency. The same stepwise procedure as above is applied for all states, red states alone, and blue states alone (Tables 3 and 4). A stepwise procedure is used to select the explanatory variables, controlling for state fixed effects, and the model is obtained from the resulting OLS regression.

Comparing performance of these models with a Newey-West estimator yield quite similar models and do not give significant improvement in the out of sample correlation, suggesting that the potential autocorrelation of residuals does not impair the performance of models estimated with robust standards errors.

### Variance decomposition

Simple variance decomposition as described below can help to quantify the contributions of each covariate to the explanatory power of the model. It follows from the equality:

$$R^{2}=Var(\sum_{i}\beta_{i}X_{i})=Cov(\sum_{i}\beta_{i}X_{i},\sum_{j}\beta_{j}X_{j})=\sum_{i}\beta_{i}.(\sum_{j}\beta_{j}Cov(X_{i},X_{j}))=\sum_{i}Contributions(X_{i})$$ (1)

Note that some contributions can be negative if the covariance terms are larger than the individual variance of variable X_i_ and are negative. This case typically occurs when a given variable is highly correlated with others but has the opposite sign. The results from the decomposition are presented in Table 5.

### Well-being in red states following the election of Barack Obama

We define red states as those states where less than 45% of the electorate voted for Obama. We estimate a simple difference in difference specification for the ten months before and the ten months after the 2008 election. We are confined to this period because the data begin in January 2008. We estimate the following equation:

$${WB}_{i,t}=\beta .({Red}_{i}*{Post}_{t})+\delta .{Post}_{t}+\pi .{Red}_{i}+\alpha_{i}+d_{t}+\varepsilon_{i,t}$$ (2)

where *WB*_*it*_ is the well-being in state *i* at time *t*, *Red*_*i*_ is a dummy variable equal to 1 if *i* is a red state, *Post*_*t*_ is a dummy equal to one if *t* occurs after the election, *α*_*i*_ is a state fixed effect and *d*_*t*_ is a vector of month dummies. The only parameter of interest is *β*, as inclusion of time and state dummies prevent the interpretation of coefficients *δ* and *π*, which are nonetheless reported to conform with the usual difference-in-difference specification. Standard errors are clustered at the state level. Results from this regression are given in Table 7.

### Mass layoff announcements and well-being

We estimate the change in well-being following the announcement of a layoff in the 16 states for which we have data. The structure of these data both demand and allow for a more flexible specification than the examination of well-being in red states following Obama’s election. As before, we use the same difference-in-difference setup, but also account to potential effects linked to the time distance to a mass layoff announcement. In particular, this refined setup allows for the fact that a mass layoff could partly be anticipated by people before its official announcement (due to media coverage), and for the fact that it could have persisting effects after the month of announcement. In practice, we use the classical framework developed by Clark et al. [28] to estimate the changes in well-being related to divorce, unemployment, and other life events at the individual level, as well as the *anticipation* and *adaptation* effects linked to those phenomenon. Our specification in this case is

$${WB}_{i,t}=\sum_{k=-10}^{10}\gamma_{k}T_{i}.\theta_{i,t,k}+\beta .T_{i}+\alpha_{i}+d_{t}+\varepsilon_{i,t}$$ (3)

where *WB*_*it*_ is the well-being in state *i* at time *t*, *T*_*i*_ is a dummy for the group of treated states (i.e. those experiencing a mass layoff), *θ_i,t,k_* is a dummy indicating that, in month t, a state i is experiencing a mass layoff at a time distance of k months (k varying from -10 months before mass layoff to +10 months after mass layoff), *α*_*i*_ are state fixed effects and d_t_ are month dummies. The estimates for parameters of interest *γ_k_* are plotted in Fig 4.

## Supporting information

S1 Fig Gallup Subjective well-being variables over time. (DOCX)

S2 Fig "Spikes" and the divorce of kim kardashian. (DOCX)

S3 Fig "Cliffs" and mace spray. (DOCX)

S4 Fig Adjustment for the January 2011 discontinuity. (DOCX)

S5 Fig Time trends in "teeth hurt". (DOCX)

S6 Fig Category variables over time. (DOCX)

S7 Fig Comparison of selected category composites to administrative data series. (DOCX)

S1 Table Category components. (DOCX)

S2 Table Bayesian model averaging results. (DOCX)
