# Correction: Well-being through the lens of the internet

**DOI:** 10.1371/journal.pone.0211586

**Published:** 2019-01-25

**Authors:** Yann Algan, Fabrice Murtin, Elizabeth Beasley, Kazuhito Higa, Claudia Senik

In the Funding section, the grant number from the funder European Research Council is missing. The correct funding information is as follows: This research was funded by CEPREMAP and the European Research Council. Yann Algan received financial support for this work from the European Commission’s Horizon 2020 program under European Research Council Consolidator Grant no. 647870. The funders had no role in study design, data collection and analysis, decision to publish, or preparation of the manuscript.

## References

[1] Algan Y, Murtin F, Beasley E, Higa K, Senik C (2019) Well-being through the lens of the internet. PLoS ONE 14(1): e0209562 10.1371/journal.pone.0209562 30633759 PMC6329518

