# Supplementary material for: Well-being through the lens of the internet
Source: PLoS One. 2019 Jan 11;14(1):e0209562. doi: 10.1371/journal.pone.0209562 (PMC6329518; doi:10.1371/journal.pone.0209562)
Supplement: S1 Fig — (DOCX) [file pone.0209562.s001.docx]

S1 Fig. Gallup Subjective Well-being Variables over time

Source : Gallup Analytics. The figure shows the evolution of the subjective well-being survey data over time.
