# Supplementary material for: Well-being through the lens of the internet
Source: PLoS One. 2019 Jan 11;14(1):e0209562. doi: 10.1371/journal.pone.0209562 (PMC6329518; doi:10.1371/journal.pone.0209562)
Supplement: S2 Fig — (DOCX) [file pone.0209562.s002.docx]

S2 Fig. "Spikes" and the Divorce of Kim Kardashian

October 31, 2011: Kim Kardashian files for divorce from Kris Humphries after 72 days of marriage

Source : Google Trends. The figure shows how high publicized events can generate spikes in the data which might potentially distort the analysis or interpretation. The spike in “divorce” related to the Kardashian divorce is unlikely to reflect an underlying increase in the searches for divorce as related to, for example, someone seeking a divorce.
