# Supplementary material for: Well-being through the lens of the internet
Source: PLoS One. 2019 Jan 11;14(1):e0209562. doi: 10.1371/journal.pone.0209562 (PMC6329518; doi:10.1371/journal.pone.0209562)
Supplement: S3 Fig — (DOCX) [file pone.0209562.s003.docx]

S3 Fig. "Cliffs" and Mace Spray

**

Source : Google Trends. The figure shows how some series of search volumes exhibit sharp increases from zero or near zero, raising concerns about using such variables in the analysis.
