# Supplementary material for: Well-being through the lens of the internet
Source: PLoS One. 2019 Jan 11;14(1):e0209562. doi: 10.1371/journal.pone.0209562 (PMC6329518; doi:10.1371/journal.pone.0209562)
Supplement: S4 Fig — (DOCX) [file pone.0209562.s004.docx]

S4 Fig. Adjustment for the January 2011 Discontinuity

Source : Google Trends. The figure shows an example of the January 2011 discontinuity, where several series have sharp jumps for reasons that are unknown(left), and demonstrates the effect of our correction procedure (right).
