# Supplementary material for: Well-being through the lens of the internet
Source: PLoS One. 2019 Jan 11;14(1):e0209562. doi: 10.1371/journal.pone.0209562 (PMC6329518; doi:10.1371/journal.pone.0209562)
Supplement: S5 Fig — (DOCX) [file pone.0209562.s005.docx]

S5 Fig. Time trends in "Teeth hurt"

Source : Google Trends. The figure shows how some search volumes exhibit striking upward trends, particularly those related to health. The difficulty with these series is that it is difficult to know whether the rise is due to an actual increase in dental pain, or whether it is due to other factors (for example, the aging of internet users, or an increase in use of the internet to obtain medical advice).
