# Supplementary material for: Well-being through the lens of the internet
Source: PLoS One. 2019 Jan 11;14(1):e0209562. doi: 10.1371/journal.pone.0209562 (PMC6329518; doi:10.1371/journal.pone.0209562)
Supplement: S6 Fig — (DOCX) [file pone.0209562.s006.docx]

S6 Fig. Category Variables over time

The figure shows how the constructed categories for evolve over time. Job Search and Job Market both show the severity of the crisis in 2008-2009 and the subsequent improvement of labor market conditions. Several of the categories exhibit sharp seasonal trends, with dips or jumps around the holidays.
