# Supplementary material for: Well-being through the lens of the internet
Source: PLoS One. 2019 Jan 11;14(1):e0209562. doi: 10.1371/journal.pone.0209562 (PMC6329518; doi:10.1371/journal.pone.0209562)
Supplement: S7 Fig — (DOCX) [file pone.0209562.s007.docx]

S7 Fig. Comparison of selected category composites to administrative data series

Source: Bureau of Labor Statistics (Unemployment Rate and Spending on Entertainment), Bankruptcy Data Project at Harvard (Chapter 11 Petitions), FBI (Intimate Crime), Mother Jones (Victims of Mass Shootings). Figure S7 provides some comparison of the categories to other social trends reflected in administrative data. Note that Job Search peaks in 2009, when the unemployment rate was increasing the most quickly, and Job Market peaks in early 2010, when the unemployment rate was stabilizing and starting to drop. Financial Security also closely tracks bankruptcy (Chapter 11) petitions in US courts. Family Life shows an increasing trend over the period, whereas Family Stress decreases after the financial crisis, and the decrease in Family Stress maps onto the decrease in Intimate Crime incidents reported by the FBI.
