# Supplementary material for: Well-being through the lens of the internet
Source: PLoS One. 2019 Jan 11;14(1):e0209562. doi: 10.1371/journal.pone.0209562 (PMC6329518; doi:10.1371/journal.pone.0209562)
Supplement: S1 Table — (DOCX) [file pone.0209562.s008.docx]

S1 Table. Category Components

| **Life Aspects** | **Category** | **Cronbach's Alpha** | **Component Keywords** |
| --- | --- | --- | --- |
| **Material Conditions** | Job Search | 0.8720 | part time job, layoffs, job fair, apprenticeships, severance pay, unemployment rate, career fair, jobs, unemployment benefits |
|  | Job Market | 0.9315 | job benefits, employment contract, career center, cover letter, pension, certification program, retirement, work experience, entry level, qualifications, discrimination, employee benefits |
|  | Financial Security | 0.9101 | 401k, banking, family budget, housing authority, section 8, inflation, student loans, school loans, interest rate, fired, financial crisis, loans, eitc, social security, coupons, medicaid, file for bankruptcy, shelter, eviction, food bank, homeless |
|  | Home Finance | 0.9618 | mortgage rates, mortgage calculator, mortgage refinancing, house price, home loan, housing crisis, mortgage payment |
|  |  |  |  |
| **Social** | Family Stress | 0.8818 | domestic abuse, marriage help, marriage problems, custody, marriage counseling, family support, womens shelter |
|  | Family Time | 0.9140 | snacks for kids, weekends, adhd, daycare, child care center, pta, kids party, volleyball, play football, reading, recipe, housework, laundry, toddler, baby shower, bullying, kids books, ideas for kids, tuition |
|  | Civic Engagement | 0.9148 | volunteering, blood donation, homeowners association, boy scouts, kiwanis, city council, freemasons, bingo, tea party, lions club, club, community meeting, civic engagement, rotary club, town council |
|  | Personal Security | 0.8657 | firearm, victims, gun, gun safety, violent crime, crime rate, assault, security camera, murder, self defense, aggression, risks, home alarm, mugging |
|  |  |  |  |
| **Health and Wellness** | Health Conditions | 0.9051 | stress, hypertension, diabetes, obesity, panic disorder, illness, tobacco use, lung cancer, heart disease, obsessive compulsive, cancer, relaxation techniques, antidepressant, health status, fracture, arthritis, asthma, relaxation, self care, sleep problems, mayo clinic, depression symptoms, symptom checker, suicide rates, drug use, chronic fatigue |
|  | Healthy Habits | 0.8441 | exercise, weights, healthy diet, dental care, fruits and vegetables, life expectancy, lose weight, health care, quit smoking |
|  | Summer Activities | 0.9417 | golf, fishing, motorcycle, ponds, hiking, biking, water sports, boating, tours, beach cottage, sightseeing, bed and breakfast, baseball, softball, play pool |
|  | Education and Ideals | 0.9862 | middle school, junior high, economics, homework, state university, moral, individualism, bill of rights, human capital, constitution, political action, social justice, dropout, reading books, grammar, tutor, buddhist, school, high school, ethics, philosophy, studies, study, mathematics, learning, skills, secondary education, writing, worship, creativity, psychologist, therapy, self esteem, morality, poverty, online courses, literature, degree, language, science, literacy, feminist, rights, relations, freedom of speech, civil rights, religion, religious, property rights, racism, governments, free speech, rituals, infant mortality, spirituality |

The table shows the internet search volumes used to construct the categories, and cronbach’s alpha calculated using seasonally adjusted search volumes.
