# Supplementary material for: Well-being through the lens of the internet
Source: PLoS One. 2019 Jan 11;14(1):e0209562. doi: 10.1371/journal.pone.0209562 (PMC6329518; doi:10.1371/journal.pone.0209562)
Supplement: S2 Table — (DOCX) [file pone.0209562.s009.docx]

S2 Table. Bayesian Model Averaging Results

| **Life Evaluation** | | **Life Evaluation in 5 Years** | | **Happiness** | | **Laugh** | | **Learn** | |
| --- | --- | --- | --- | --- | --- | --- | --- | --- | --- |
| Category | PIP | Category | PIP | Category | PIP | Category | PIP | Category | PIP |
| Job Search | 1 | Family Stress | .9999999 | Family Life | .9999308 | Job Search | 1 | Job Search | 1 |
| Family Stress | .9999345 | Financial Security | .9999994 | Job Market | .9988018 | Family Life | 1 | Family Life | .9999999 |
| Job Market | .9959486 | Job Search | .9987293 | Financial Security | .9612713 | Personal Security | .9987682 | Health Conditions | .9926389 |
| Healthy Habits | .9756049 | Personal Security | .9867251 | Job Search | .7824603 | Summer Leisure | .9339863 | Financial Security | .9868134 |
| Civic Engagement | .896345 | Job Market | .9126053 | Summer Leisure | .5180083 | Health Conditions | .9287642 | Healthy Habits | .8700904 |
| Financial Security | .4405012 | Family Life | .7997323 | Education and Ideals | .3740062 | Financial Security | .7978849 | Job Market | .7783921 |
| Summer Leisure | .0798991 | Education and Ideals | .1033073 | Family Stress | .2125029 | Civic Engagement | .5212126 | Family Stress | .2353987 |
|  |  | Civic Engagement | .0968726 | Civic Engagement | .1982485 | Job Market | .4633149 | Summer Leisure | .136816 |
|  |  | Summer Leisure | .0882474 | Health Conditions | .0954218 | Family Stress | .1317561 | Education and Ideals | .0983368 |
|  |  | Health Conditions | .0730709 | Personal Security | .0713978 | Education and Ideals | .1096197 | Civic Engagement | .0924329 |
|  |  |  |  |  |  |  |  | Personal Security | .0689583 |
| **Respect** | | **Anger** | | **Stress** | | **Worry** | | **Sadness** | |
| Category | PIP | Category | PIP | Category | PIP | Category | PIP | Category | PIP |
| Job Market | 1 | Job Search | 1 | Family Life | 1 | Job Search | 1 | Job Search | 1 |
| Job Search | .9999644 | Financial Security | .9999973 | Job Search | 1 | Summer Leisure | .9999992 | Personal Security | .9999999 |
| Financial Security | .9995359 | Summer Leisure | .9999353 | Healthy Habits | .9999991 | Healthy Habits | .9999803 | Family Life | .9998972 |
| Healthy Habits | .2881511 | Education and Ideals | .9795223 | Summer Leisure | .9999747 | Financial Security | .9998447 | Summer Leisure | .9981101 |
| Personal Security | .1305427 | Civic Engagement | .0981886 | Financial Security | .9726784 | Family Stress | .6311848 | Education and Ideals | .9921088 |
| Family Stress | .1250909 | Health Conditions | .0951104 | Education and Ideals | .7365542 | Education and Ideals | .5506082 | Financial Security | .9878952 |
| Summer Leisure | .0748373 | Job Market | .0887169 | Civic Engagement | .4862978 | Health Conditions | .5280867 | Healthy Habits | .9852301 |
|  |  | Personal Security | .0664031 | Health Conditions | .3005131 | Personal Security | .5102105 | Health Conditions | .1115238 |
|  |  | Family Stress | .0663557 | Family Stress | .2364499 | Civic Engagement | .0752305 | Civic Engagement | .0924093 |
|  |  |  |  | Personal Security | .1218278 | Family Life | .067823 | Family Stress | .0690964 |
|  |  |  |  | Job Market | .0749033 |  |  |  |  |
| Output from Bayesian Model Selection routine carried out in R using BMS package, with 1M iterations. Fixed effects were included for weeks during the months of December and January, and months during the rest of the year. PIP indicates the posterior inclusion probability. To avoid overfitting, the categories used for each SWB variable were restricted to those that increased the adjusted R2 when included in a regression with month and week controls. | | | | | | | | | |
